# Kidney glycolysis serves as a mammalian phosphate sensor that maintains phosphate homeostasis

**DOI:** 10.1172/JCI164610

**Published:** 2023-04-17

**Authors:** Wen Zhou, Petra Simic, Iris Y. Zhou, Peter Caravan, Xavier Vela Parada, Donghai Wen, Onica L. Washington, Maria Shvedova, Kerry A. Pierce, Clary B. Clish, Michael Mannstadt, Tatsuya Kobayashi, Marc N. Wein, Harald Jüppner, Eugene P. Rhee

**Affiliations:** 1 Nephrology Division, Department of Medicine, and; 2 Endocrine Unit, Department of Medicine, Massachusetts General Hospital, Boston, Massachusetts, USA.; 3 Athinoula A. Martinos Center for Biomedical Imaging, Department of Radiology, Massachusetts General Hospital, Charlestown, Massachusetts, USA.; 4 Broad Institute of MIT and Harvard, Cambridge, Massachusetts, USA.; 5 Pediatric Nephrology Unit, Department of Pediatrics, Massachusetts General Hospital, Boston, Massachusetts, USA.

**Keywords:** Endocrinology, Nephrology, Calcium, Chronic kidney disease, Glucose metabolism

## Abstract

How phosphate levels are detected in mammals is unknown. The bone-derived hormone fibroblast growth factor 23 (FGF23) lowers blood phosphate levels by reducing kidney phosphate reabsorption and 1,25(OH)_2_D production, but phosphate does not directly stimulate bone FGF23 expression. Using PET scanning and LC-MS, we found that phosphate increases kidney-specific glycolysis and synthesis of glycerol-3-phosphate (G-3-P), which then circulates to bone to trigger FGF23 production. Further, we found that G-3-P dehydrogenase 1 (Gpd1), a cytosolic enzyme that synthesizes G-3-P and oxidizes NADH to NAD+, is required for phosphate-stimulated G-3-P and FGF23 production and prevention of hyperphosphatemia. In proximal tubule cells, we found that phosphate availability is substrate-limiting for glycolysis and G-3-P production and that increased glycolysis and Gpd1 activity are coupled through cytosolic NAD+ recycling. Finally, we show that the type II sodium-dependent phosphate cotransporter Npt2a, which is primarily expressed in the proximal tubule, conferred kidney specificity to phosphate-stimulated G-3-P production. Importantly, exogenous G-3-P stimulated FGF23 production when Npt2a or Gpd1 were absent, confirming that it was the key circulating factor downstream of glycolytic phosphate sensing in the kidney. Together, these findings place glycolysis at the nexus of mineral and energy metabolism and identify a kidney-bone feedback loop that controls phosphate homeostasis.

## Graphical abstract

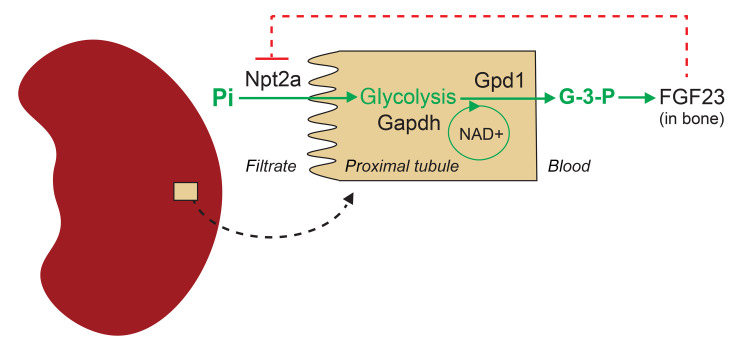

## Introduction

Phosphate is essential for life, with a fundamental role in bone mineralization, cell signaling, synthesis of lipids and nucleic acids, and energy metabolism. Bacteria and yeast sense inorganic phosphate (Pi) with a multi-protein membrane complex that modulates genes important for Pi uptake and metabolism, but no mammalian ortholog has been identified (1). Recently, Pi was shown to bind to the calcium-sensing receptor (CaSR) on parathyroid cells to stimulate parathyroid hormone (PTH) secretion (2). Although PTH has a range of effects on Pi, its primary purpose is maintenance of extracellular ionized calcium levels. Thus, the sensor dedicated primarily to mammalian Pi homeostasis remains unknown.

Archetypal biosensors act upstream of circulating factors to maintain systemic homeostasis. For example, oxygen, glucose, and calcium sensors trigger the synthesis of erythropoietin, insulin, and PTH (3–5). Fibroblast growth factor 23 (FGF23) is a bone-derived hormone, identified in the year 2000 as the cause of autosomal dominant hypophosphatemic rickets (6). This and other genetic causes of FGF23 excess and deficiency cause severe hypo- and hyperphosphatemia, respectively (7). FGF23 has 2 major effects; like PTH, it reduces kidney Pi reabsorption by downregulating proximal tubular expression of the Npt2a/2c sodium-phosphate cotransporters, and, unlike PTH, it reduces kidney 1,25-dihydroxyvitamin D [1,25(OH)_2_D] synthesis, thus reducing intestinal Pi uptake. Although Pi may reduce FGF23 cleavage in bone (8), it does not directly stimulate bone FGF23 gene expression, and Pi loading in animals or humans results in a delayed rise in FGF23 (9). These findings raise the possibility that the Pi sensor upstream of FGF23 is extraskeletal.

Recently, we identified a signaling axis whereby kidney-derived glycerol-3-phosphate (G-3-P), a byproduct of glycolysis, circulates to bone and triggers FGF23 expression in the nonphysiologic context of acute kidney injury (10). This study began with an unbiased metabolomic and proteomic screen of kidney venous samples obtained via invasive catheterization from 17 people. Of the more than 5,000 molecules examined, G-3-P was the top hit, demonstrating a striking correlation with arterial intact FGF23. This study used renal ischemia to show that increased glycolysis and G-3-P production in the kidney are coupled. Notably, Pi is a key substrate in glycolysis at the step catalyzed by glyceraldehyde 3-phosphate dehydrogenase (Gapdh). This reaction has been shown to control the rate of glycolysis under certain conditions (11, 12), with intracellular Pi availability proposed as a key factor (13). However, whether exogenous Pi can trigger kidney glycolysis and G-3-P production, and thus modulate systemic FGF23 and Pi homeostasis, has not been examined.

## Results

### Pi stimulates kidney-specific glycolysis and G-3-P production.

**Figure 1:**
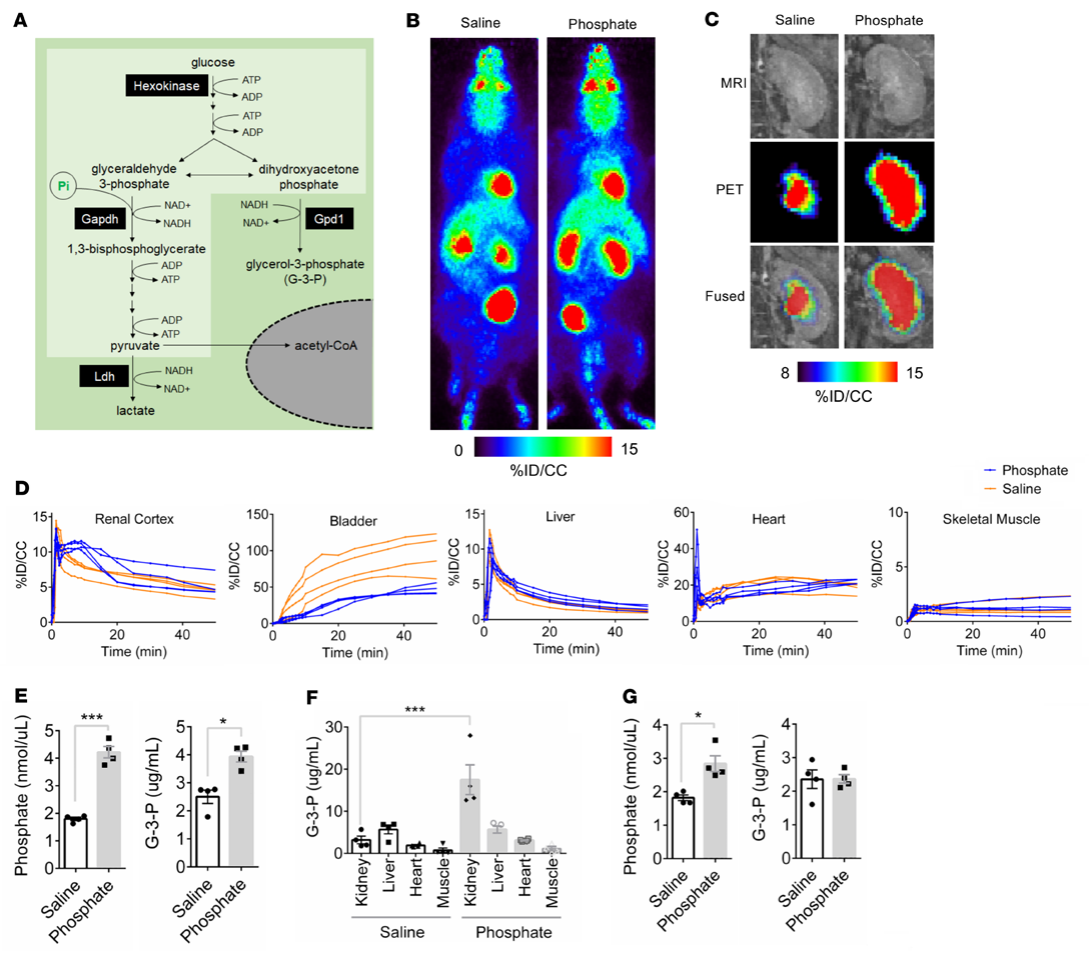
Phosphate stimulates kidney-specific glycolysis and G-3-P production. (**A**) Schematic illustration of Pi’s entry into glycolysis (light green) and G-3-P biosynthesis. (**B** and **C**) Representative whole–animal maximum intensity projection PET scan images (**B**) and kidney coronal MRI, PET, and fused images (**C**) 10 minutes after i.v. ^18^F-FDG and either sodium phosphate or sodium chloride. (**D**) ^18^F-FDG signal expressed as percent injected dose per cubic centimeter of tissue (%ID/CC) in renal cortex, bladder, liver, heart, and skeletal muscle following i.v. ^18^F-FDG and either sodium phosphate or sodium chloride in C57BL/6J mice (*n* = 4 per group). (**E** and **F**) Blood phosphate and G-3-P concentrations (**E**) and tissue G-3-P concentrations (**F**) 10 minutes after i.v. sodium phosphate or sodium chloride in C57BL/6J mice (*n* = 4 per group). (**G**) Blood phosphate and G-3-P concentrations 10 minutes after i.v. sodium phosphate or sodium chloride in C57BL/6J mice that were pretreated with empagliflozin 10 mg/kg by oral gavage 2 hours before injections (*n* = 4 per group). Values are mean ± SEM. *P < 0.05, ***P < 0.0001. Unpaired Student’s *t* test (**E** and **G**) or ANOVA with Tukey’s multiple comparisons test (**F**).

As shown in Figure 1A, Pi is a substrate for a key, irreversible step in glycolysis catalyzed by Gapdh, raising the question of whether Pi may be substrate-limiting for glycolysis and G-3-P production. 18F-fluorodeoxyglucose (^18^F-FDG) enters glycolysis at the first committed step catalyzed by hexokinase, but then cannot be further metabolized because of an absent hydroxyl group. Using ^18^F-FDG PET scanning, we observed a significant increase in ^18^F-FDG signal in the kidney cortex for approximately 20 minutes following the administration of i.v. sodium Pi (6 mg) compared with equimolar saline (2.7 mg) in C57BL/6J mice (Figures 1, B–D, and Supplemental Figure 1A; supplemental material available online with this article; https://doi.org/10.1172/JCI164610DS1). Notably, this increase in kidney ^18^F-FDG signal was mirrored by a marked reduction in ^18^F-FDG signal in the bladder, demonstrating reduced urinary excretion of ^18^F-FDG in animals administered sodium Pi. No difference in ^18^F-FDG signal was observed in heart, liver, or skeletal muscle following administration of sodium Pi versus saline (Figure 1D).

To assess whether this kidney-specific increase in Pi-stimulated glycolysis was coupled to G-3-P production, we collected plasma and kidney, heart, liver, and skeletal muscle tissue from mice 10 minutes after i.v. administration of sodium Pi or saline. We confirmed a significant increase in G-3-P in plasma and kidney tissue (Figure 1, E and F), with no changes observed in other tissues (Figure 1F); the approximately 5.5-fold increase in kidney G-3-P level with sodium Pi was accompanied by an approximately 1.3-fold increase in the kidney lactate level(Supplemental Figure 1B). By contrast, sodium Pi did not increase blood G-3-P levels in C57BL/6J mice predosed with empagliflozin, an inhibitor of glucose uptake in the kidney proximal tubule (Figure 1G). Further, i.v. Pi had no impact on circulating G-3-P concentrations in mice that had undergone bilateral clamping of the renal arteries and veins, confirming that the kidney is the major source of Pi-stimulated G-3-P production (Supplemental Figure 1C). Finally, we found that blood G-3-P levels increase within 1 to 2 hours following oral Pi ingestion in healthy people (Supplemental Figure 1, D and E).

### Pi-stimulated G-3-P production requires glycolysis.

**Figure 2:**
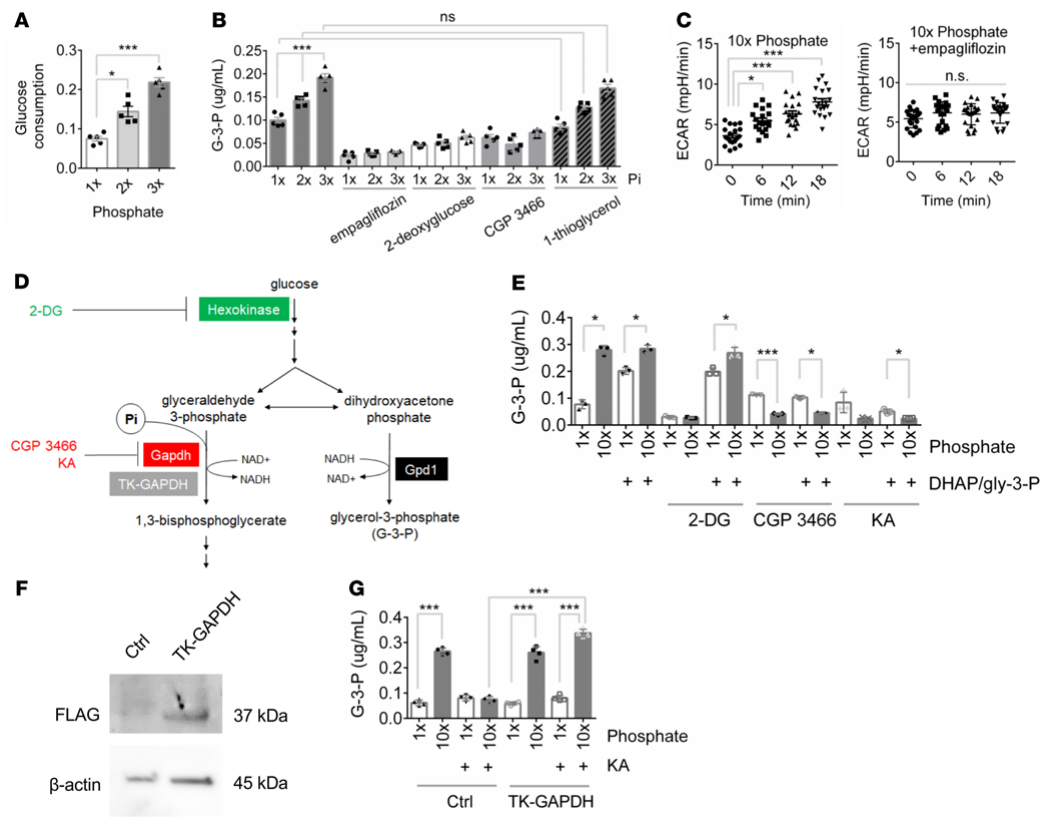
Phosphate-stimulated G-3-P production requires glycolysis. (**A**) Media glucose consumption 6 hours after primary human proximal tubule cells were incubated with increasing concentrations of sodium phosphate (*n* = 5 per group). (**B**) Media G-3-P concentrations 6 hours after primary human proximal tubule cells were incubated with increasing concentrations of sodium Pi, ± empagliflozin (1 μM), 2-deoxyglucose (25 mM), CGP 3466 (5 μM), and 1-thioglycerol (10 mM) (*n* = 5 per group). (**C**) Extracellular acidification measured by Seahorse in OK cells treated with high phosphate ± empagliflozin (1 μM) (*n* = 16 per group). (**D**) Schema of hexokinase and Gapdh inhibition and rescue experiments. (**E**) Media G-3-P concentration 120 minutes after OK cells were incubated with 1 × or 10 × sodium phosphate, with or without 2-deoxyglucose (2-DG, 25 mM), CGP 3466 (5 μM), or koningic acid (KA; 33 μM), and with or without a mixture of 1 mM each dihydroxyacetone phosphate and glyceraldehyde-3-phosphate (DHAP/gly 3-P) (*n* = 3 per group). (**F**) Immunoblot of FLAG and β-actin in OK cells transfected with FLAG-TK-GAPDH or control vector. (**G**) Media G-3-P concentration at 120 minutes from OK cells transfected with TK-GAPDH or control vector incubated with 1 × or 10 × sodium phosphate, with or without KA (33 μM) (*n* = 4 per group). 1 × Pi = 0.9 mM. Values are mean ± SEM. *P < 0.05, ***P < 0.0001. ANOVA with Dunnett’s multiple comparisons test (**A**), ANOVA with Tukey’s multiple comparisons test (**B**, **C**, **E**, and **G**).

Using primary human proximal tubule cells, we found that Pi, but not sulfate, stimulated a dose-dependent increase in glucose consumption and G-3-P secretion (Figure 2, A and B and Supplemental Figure 2, A and B). Pi’s effect on G-3-P production in these cells was abrogated by the coadministration of empagliflozin; 2-deoxyglucose, a hexokinase inhibitor; and CGP 3466, a Gapdh inhibitor (Figure 2B) (14). By contrast, Pi-stimulated G-3-P production was unaffected by 1-thioglycerol, an inhibitor of glycerol kinase (Figure 2B) (15). Modifying media glucose or calcium did not have a marked effect on G-3-P production (Supplemental Figure 2, C and D). All key findings were replicated in opossum proximal tubular (OK) cells (Supplemental Figure 2, E–H) (16, 17), with additional corroboration of the inhibitory effect of empagliflozin on Pi-stimulated glycolysis by real-time measurement of extracellular acidification (lactate secretion) (Figure 2C and Supplemental Figure 2I).

Importantly, we found that the addition of a mixture of glyceraldehyde-3-phosphate and dihydroxyacetone phosphate was able to rescue Pi-stimulated G-3-P production in OK cells treated with 2-deoxyglucose (Figure 2, D and E). That is, hexokinase activity is not required for Pi-stimulated G-3-P production as long as downstream metabolites are available. By contrast, glyceraldehyde-3-phosphate and dihydroxyacetone phosphate were unable to rescue Pi-stimulated G-3-P production in OK cells treated with CGP 3466 or koningic acid, an additional Gapdh inhibitor (Figure 2E). Thus, Gapdh activity is required for Pi to stimulate G-3-P production, even when the G-3-P precursor dihydroxyacetone phosphate is provided. Further, expression of a koningic acid–resistant Gapdh isolated from *T*. *koningii* (TK-GAPDH) restored Pi-stimulated G-3-P production in koningic acid–treated cells, confirming the requirement for Gapdh activity in mediating its substrate’s, Pi, effect on G-3-P production (Figure 2, F and G) (18, 19). Pi did not stimulate G-3-P secretion in cultured osteocytes (Supplemental Figure 2J).

### Pi stimulates G-3-P production in the fed state, without systematic changes in metabolic gene expression.

The kidney proximal tubule upregulates gluconeogenesis during fasting and metabolic acidosis (20). Notably, all of the in vivo experiments described to this point were conducted in fed mice. To test whether Pi-stimulated G-3-P production was impacted by fasting, we administered i.v. sodium Pi (6mg) or equimolar saline (2.7mg) to C57BL/6J mice that were fasted for 12 hours and observed an attenuated response in blood G-3-P (Supplemental Figure 3, A and B). We further examined the effect of glycolytic versus gluconeogenic conditions in vitro and found that a combination of glucose- and insulin-free media and supplemental lactate caused OK cells to secrete glucose; under these conditions, Pi had no effect on G-3-P secretion, in sharp contrast to when exogenous glucose was provided (Supplemental Figure 3, C and D).

Next, we performed RNA-Seq of mRNA obtained from the kidney cortex of C57BL/6J mice after 3 days on a normal Pi (0.6%) or high Pi (1.2%) diet. Aside from a modest increase in expression of the glycolytic gene *Pklr*, which encodes Pyruvate kinase L/R (log2 = 0.34, Q = 0.045), no other significant changes in gene expression in glycolysis or gluconeogenesis were observed (Supplemental Figure 3E). There was also no change in the expression of more than 40 genes involved in fatty oxidation (Supplemental Figure 3F), G-3-P dehydrogenase 1 (*Gpd1;* log2 = 0.13, Q = 0.62), G-3-P dehydrogenase 2 (*Gpd2*; log2 = 0.16, Q = 0.93), or glycerol kinase (*Gk*; log2 = –0.0039, Q = 0.99) on high Pi diet. Finally, we found that i.v. and dietary Pi loading were not associated with a significant acid load and did not enhance kidney ammoniagenesis (Supplemental Figure 4). Thus, Pi stimulated kidney glycolysis in the physiologically relevant context of feeding — when both glucose and Pi were ingested — and enhanced glycolytic activity without systematic changes in metabolic gene expression.

### Gpd1 mediates Pi-stimulated G-3-P and FGF23 production to regulate systemic Pi homeostasis.

**Figure 3:**
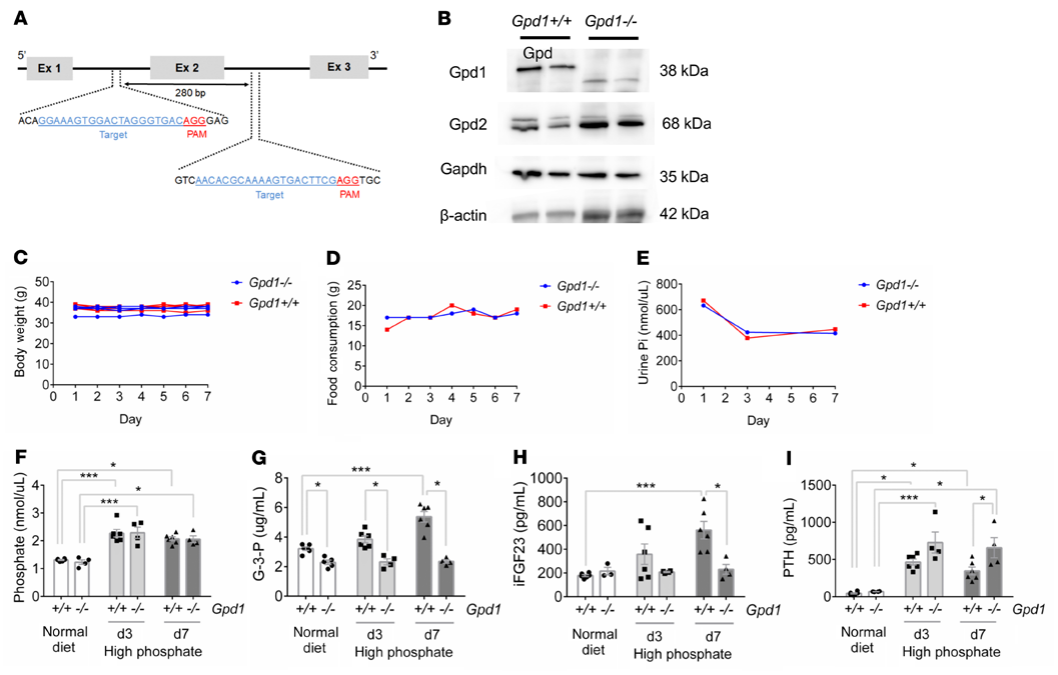
Gpd1 mediates phosphate-stimulated G-3-P and FGF23 production. (**A**) Schema for targeted deletion of Gpd1. (**B**) Immunoblot of Gpd1, Gpd2, Gapdh, and β-actin in kidney tissue from *Gpd1^+/+^* and *Gpd1^–/–^* mice. (**C**–**E**) Body weight (**C**), food consumption (**D**), and urine Pi (**E**) from *Gpd1^+/+^* and *Gpd1^–/–^* mice on high phosphate diet (1.2%) for 7 days (note, food consumption and urine Pi were assessed per cage, *n* = 4 mice per diet group). (**F**–**I**) Blood phosphate (**F**), G-3-P (**G**), intact FGF23 (iFGF23) (**H**), and PTH (**I**) concentrations in *Gpd1^+/+^* and *Gpd1^–/–^* mice fed a normal diet (0.6% Pi) and after 3 and 7 days on high phosphate diet (1.2% Pi) (*n* = 3–6 per group). Values are mean ± SEM. *P < 0.05, ***P < 0.0001. Unpaired student’s *t* test (**F**–**I**).

Gpd1 is a cytosolic enzyme that converts the glycolytic intermediate dihydroxyacetone phosphate to G-3-P and oxidizes NADH back to NAD+. An inbred mouse strain with a spontaneous *Gpd1* mutation resulting in absent enzyme expression has previously been described as viable and fertile (21). In addition, these mice were noted to have a buildup of glycolytic intermediates up to the Gapdh step and reduction of metabolites downstream of Gapdh in muscle, suggesting that Gpd1 deficiency causes a block in glycolysis at Gapdh (22). Therefore, we generated *Gpd1^–/–^* mice to examine the role of Gpd1 in Pi-stimulated G-3-P production (Figure 3, A and B and Supplemental Figure 5A). No difference was observed between *Gpd1^–/–^* mice and *Gpd1^+/+^* littermates in body weight, food intake, or urinary Pi (Figure 3, C–E). On a normal Pi diet (0.6%), G-3-P levels were lower in *Gpd1^–/–^* mice than in their *Gpd1^+/+^* littermates, but no difference was observed in Pi or FGF23 (Figure 3, F–H); in addition, no difference was observed in calcium or kidney function, but *Gpd1^–/–^* mice did have higher 1,25(OH)_2_D levels (Supplemental Figure 5, B–E). As with i.v. Pi administration, an increase in dietary Pi content from 0.6% to 1.2% increased blood Pi and G-3-P concentrations in *Gpd1^+/+^* mice, with a concomitant increase in intact FGF23 levels. By contrast, dietary Pi loading increased blood Pi concentrations, but had no effect on blood G-3-P or FGF23 levels in *Gpd1^–/–^* mice (Figure 3, F–H). Despite the significant difference in FGF23 levels, no difference in blood Pi concentration was observed between *Gpd1^–/–^* mice and their *Gpd1^+/+^* littermates in response to the high Pi diet (Figure 3F), likely because of a compensatory increase in PTH in *Gpd1^–/–^* mice (Figure 3I).

**Figure 4:**
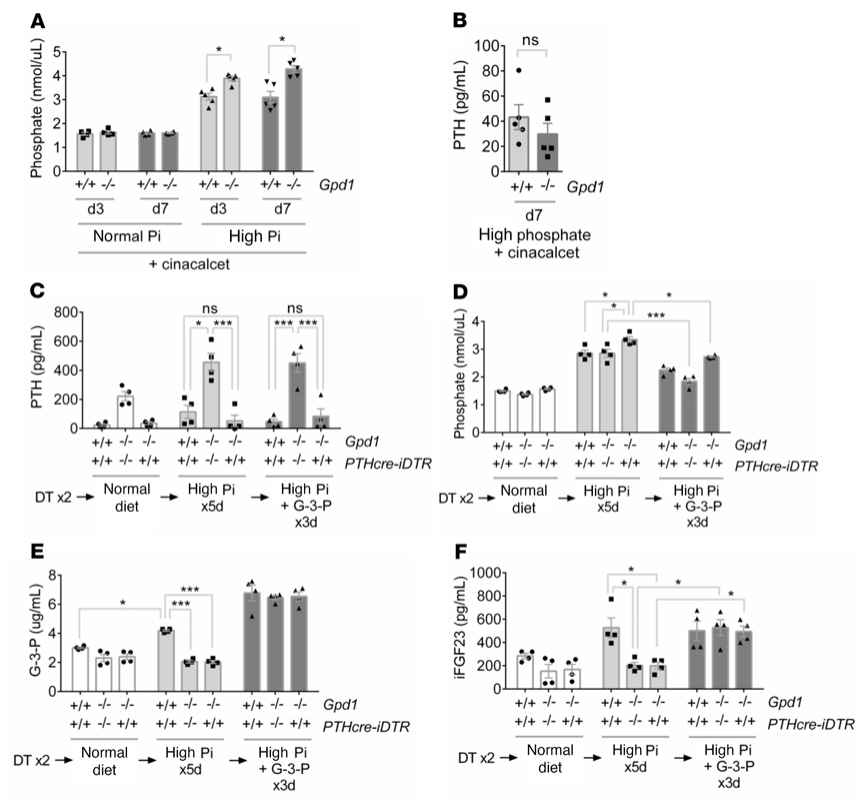
*Gpd1^–/–^* mice with induced hypoparathyroidism develop hyperphosphatemia that is rescued by G-3-P. (**A**) Blood phosphate concentrations in *Gpd1^+/+^* and *Gpd1^–/–^* mice fed a normal (0.6% Pi) or high phosphate (1.2% Pi) diet supplemented with cinacalcet for 3 and 7 days (*n* = 5 per group). (**B**) Blood PTH concentrations in *Gpd1^+/+^* and *Gpd1^–/–^* mice fed a high phosphate diet supplemented with cinacalcet for 7 days (*n* = 5 per group). (**C**–**F**) Blood PTH (**C**), phosphate (**D**), G-3-P (**E**), and intact FGF23 (iFGF23) (**F**) concentrations in DT–treated *Gpd1^+/+^*-PTHcre-iDTR, *Gpd1^–/–^*, and *Gpd1^–/–^*-PTHcre-iDTR mice on a normal diet, after 5 days on a high phosphate diet, and after an additional 3 days on a high phosphate diet plus daily i.p. G-3-P (300 mg/kg) (*n* = 4 per group). Values are mean ± SEM. *P < 0.05, ***P < 0.0001. Unpaired student’s *t* test (**A** and **B**) or ANOVA with Tukey’s multiple comparisons test (**C**–**F**).

We used 2 approaches to remove the compensatory effect of PTH on Pi regulation. First, we administered cinacalcet, a calcimimetic that reduces PTH production, and found that this yielded significantly higher Pi concentrations in *Gpd1^–/–^* mice compared with *Gpd1^+/+^* littermates on high Pi diet (Figure 4, A and B). Second, we administered diphtheria toxin (DT) to *Gpd1^–/–^* and *Gpd1^+/+^* mice with parathyroid-specific expression of the DT receptor to induce hypoparathyroidism (Figure 4C) (23). In DT-injected animals on a high Pi diet, we observed significantly higher Pi concentrations in *Gpd1^–/–^*-PTHcre-iDTR mice compared with *Gpd1^+/+^*-PTHcre-iDTR mice and *Gpd1^–/–^* littermates that did not express the DT receptor (Figure 4D). Importantly, administration of exogenous G-3-P to the DT–treated *Gpd1^–/–^*-PTHcre-iDTR mice increased FGF23 levels and reduced blood Pi concentrations back to levels observed in *Gpd1^+/+^*-PTHcre-iDTR mice (Figure 4, D–F). Together, these findings establish the role of Gpd1 in Pi-stimulated G-3-P production, subsequent synthesis of FGF23, and systemic Pi homeostasis in vivo.

### Phosphate-stimulated glycolysis and Gpd1 activity are coupled through cytosolic NAD+ recycling.

**Figure 5:**
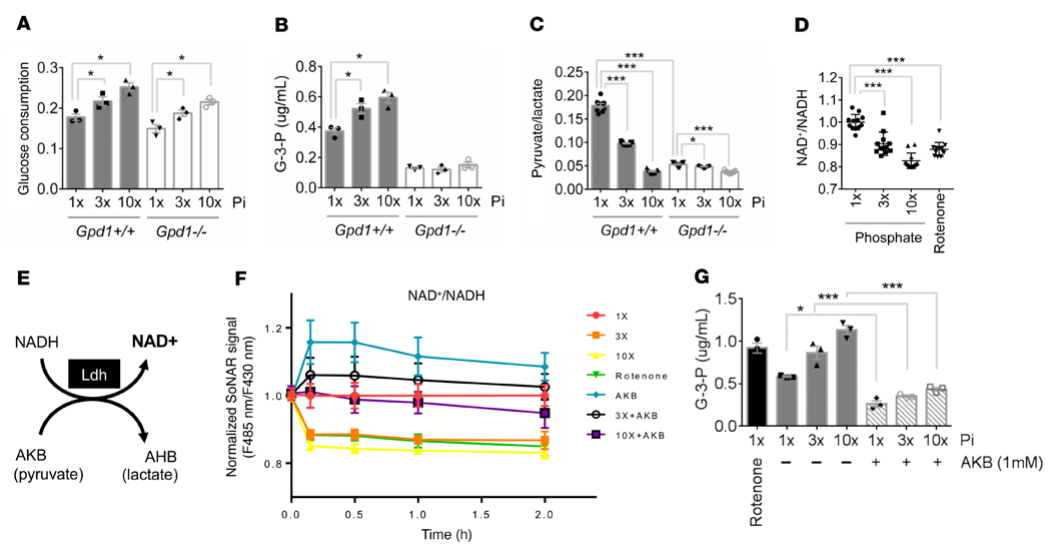
Phosphate-stimulated glycolysis and Gpd1 activity are coupled through cytosolic NAD+ recycling. (**A**–**C**) Media glucose consumption (**A**), media G-3-P concentration (**B**), and media pyruvate/lactate ratio (**C**) from primary proximal tubular cells isolated from *Gpd1^+/+^* and *Gpd1^–/–^* mice treated for 6 hours with increasing concentrations of phosphate (Pi) (*n* = 3 per group, with 3 technical replicates per sample for **C**). (**D**) Cytosolic NAD+/NADH measured in SoNar-expressing OK cells treated with rotenone (0.5 μM) or increasing concentrations of phosphate for 2 hours (*n* = 12 per group). (**E**) Schema for LDH-mediated NAD+ recycling; AKB, α-ketobutyrate; AHB, α-hydroxybutyrate. (**F**) Cytosolic NAD+/NADH measured with SoNar in OK cells treated with rotenone or increasing concentrations of phosphate ± AKB (1 mM) (*n* = 3 per group). (**G**) Media G-3-P concentrations from OK cells treated with rotenone (0.5 μM) or increasing concentrations of phosphate ± AKB (1 mM) for 2 hours (*n* = 3 per group). 1 × Pi = 0.9mM. Values are mean ± SEM. *P < 0.05, ***P < 0.0001. ANOVA with Dunnett’s multiple comparisons test (**A**–**D**) or ANOVA with Tukey’s multiple comparisons test (**G**).

The reactions catalyzed by Gapdh and Gpd1 act in parallel, not in series, raising the question of why a Pi-stimulated increase in Gapdh activity also increased Gpd1-mediated G-3-P production. In glycolysis, glucose metabolism is coupled to the reduction of cytosolic NAD+ to NADH. The flow of electrons from cytosolic NADH to an available electron acceptor restores cytosolic NAD+ and permits ongoing glycolysis. To test a potential role for Gpd1-mediated NAD+ regeneration in Pi-stimulated G-3-P production, we examined primary proximal tubular cells isolated from *Gpd1^–/–^* and *Gpd1^+/+^* mice. As with primary human proximal tubule and OK cells, Pi stimulated an increase in glucose consumption and G-3-P production in *Gpd1^+/+^* primary proximal tubular cells (Figure 5, A and B). By contrast, Pi stimulated an increase in glucose consumption but had no effect on G-3-P production in *Gpd1^–/–^* cells. Under basal conditions, the media pyruvate/lactate ratio, a surrogate for cytosolic NAD+/NADH (24), was reduced in *Gpd1^–/–^* cells compared to *Gpd1^+/+^* cells (Figure 5C), consistent with a role for Gpd1 in cytosolic NAD+ recycling. In both, the addition of Pi further decreased the media pyruvate/lactate ratio, which was as expected with increased glycolysis.

Next, we transfected OK cells with SoNAR, a genetically encoded fluorescent sensor that permits real-time assessment of cytosolic NAD+/NADH ratio (25). Consistent with the media pyruvate/lactate results in primary mouse cell culture, Pi supplementation lowered directly measured cytosolic NAD+/NADH in OK cells (Figure 5D). α-ketobutyrate (AKB) is a lactate dehydrogenase (LDH) substrate that can increase the cytosolic NAD+/NADH ratio without contributing carbon molecules to metabolism (Figure 5E) (26). We found that increasing LDH activity with AKB prevented the reduction in cytosolic NAD+/NADH following supplemental Pi, and, further, abrogated Pi-stimulated G-3-P production (Figure 5, F and G). Together, these findings identified cytosolic NAD+ availability as a key biochemical link between Gapdh and Gpd1 activity and aligned with recent work highlighting the cytosolic NAD+/NADH ratio as an important causal mediator in cellular metabolism (27–30).

### Npt2a inhibition reduces G-3-P production in vitro and in vivo.

**Figure 6:**
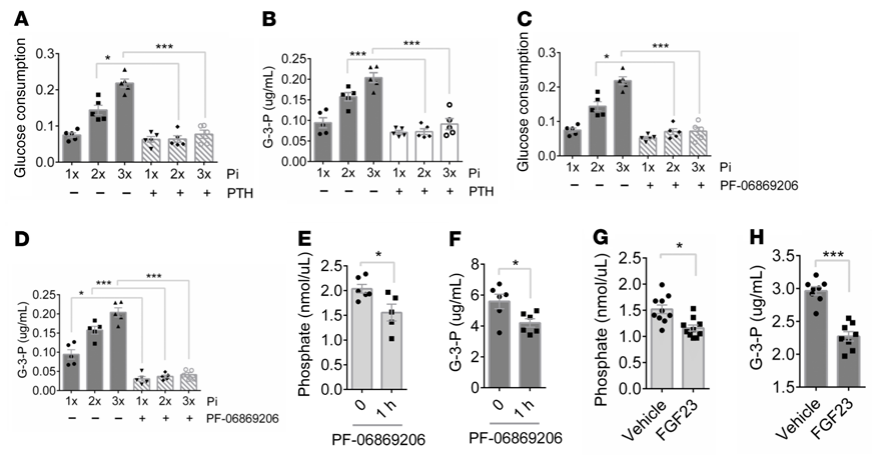
Npt2a inhibition reduces G-3-P production in vitro and in vivo. (**A** and **B**) Media glucose consumption (**A**) and G-3-P concentration (**B**) from primary human proximal tubule cells treated with increasing concentrations of Pi ± PTH (0.1 μM) for 6 hours (*n* = 5 per group). (**C** and **D**) Media glucose consumption (**C**) and G-3-P concentration (**D**) from primary human proximal tubule cells treated with phosphate ± PF-06869206 (10 μM) for 6 hours (*n* = 5 per group). (**E** and **F**) Blood phosphate (**E**) and G-3-P concentrations (**F**) in C57BL/6J mice before and 1 hour after PF-06869206 dosing (300 mg/kg) (*n* = 5–6 per group). (**G** and **H**) Blood phosphate (**G**) and G-3-P concentrations (**H**) in C57BL/6J mice 12 hours after i.p. recombinant human FGF23 (5 μg) or vehicle (*n* = 8 per group). 1 × Pi = 0.9mM. Values are mean ± SEM. *P < 0.05, ***P < 0.0001. ANOVA with Tukey’s multiple comparisons test (**A**–**D**) or unpaired student’s *t* test (**E**–**H**).

Whereas glycolysis and Gpd1 are ubiquitous, the type II sodium-phosphate cotransporters (Npt2a and Npt2c) are primarily expressed in the kidney proximal tubule. Humans and mice deficient in Npt2a, which makes the dominant contribution to renal Pi reabsorption, have urinary-Pi wasting and low blood–Pi concentrations (31–33). To assess whether Npt2a may confer kidney specificity to Pi-stimulated Gpd1 activity, we first examined the effect of reduced Npt2a action on Pi-stimulated G-3-P production in vitro. We found that pretreatment with PTH, which reduced tubular cell Npt2a (and Npt2c) expression (17, 34), significantly attenuated Pi-stimulated glucose consumption and G-3-P production in primary human proximal tubule cells and OK cells (Figure 6, A and B and Supplemental Figure 6, A and B). Similarly, we found that PF-06869206, a small molecule Npt2a inhibitor (35), also attenuated Pi-stimulated glucose consumption and G-3-P production in these cells (Figure 6, C and D and Supplemental Figure 6, C and D), reinforcing the view that intracellular Pi is substrate-limiting for proximal tubular cell glycolysis and G-3-P production. To corroborate these findings in vivo, we dosed C57BL/6J mice with a single dose of PF-06869206 by oral gavage, sampling blood immediately before and 1 hour after dosing. As shown in Figure 6, E and F, the drop in blood Pi following inhibition of proximal tubule Pi reabsorption acutely lowered circulating G-3-P. Similarly, we found that the drop in blood Pi following administration of recombinant FGF23 also resulted in reduced blood G-3-P levels (Figure 6, G and H).

### Npt2a is required for kidney glycolytic Pi sensing.

**Figure 7:**
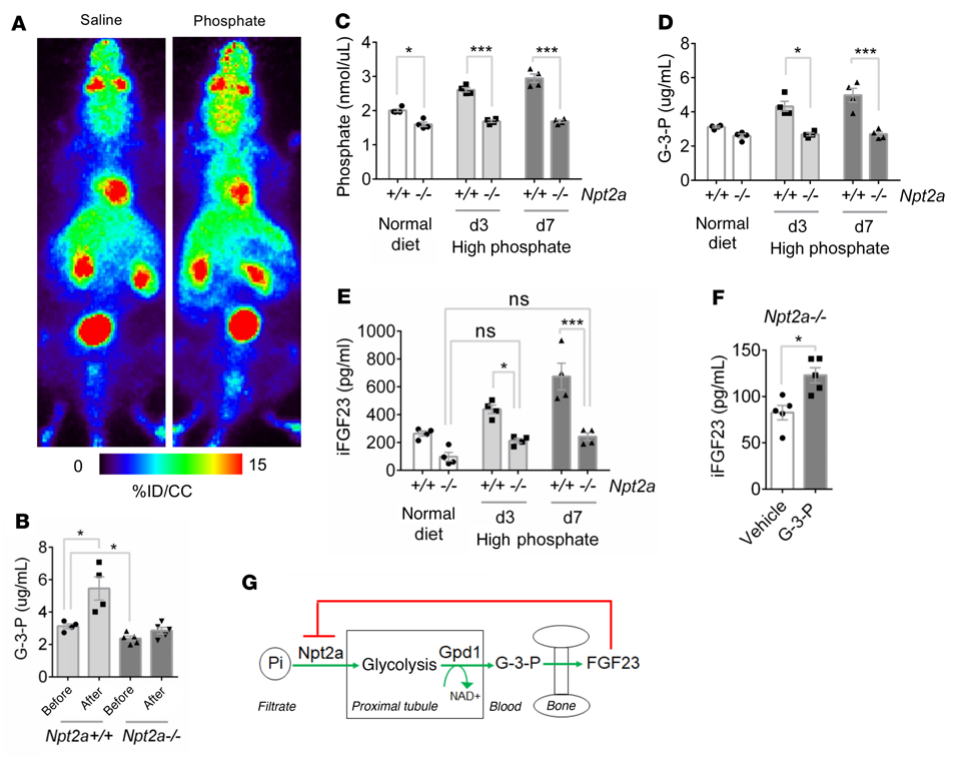
Npt2a is required for kidney glycolytic phosphate sensing. (**A**) Representative whole-animal maximum–intensity projection PET scan images 10 minutes after i.v. ^18^F-FDG and either sodium phosphate or sodium chloride injection of *Npt2a^–/–^* mice. (**B**) Blood G-3-P concentration in *Npt2a^+/+^* and *Npt2a^–/–^* mice before and 10 minutes after i.v. sodium phosphate (*n* = 4–5 per group). (**C**–**E**) Blood phosphate (C), G-3-P (D), and intact FGF23 (iFGF23) (**E**) concentrations in *Npt2a^+/+^* and *Npt2a^–/–^* mice fed a normal diet (0.6% Pi) and after 3 and 7 days on high phosphate diet (1.2% Pi) (*n* = 4 per group). (**F**) Blood iFGF23 concentration in *Npt2a^–/–^* mice 24 hours after i.p. G-3-P (300 mg/kg) or vehicle (*n* = 5 per group). (**G**) Schema showing feedback loop with Npt2a mediated phosphate uptake, proximal tubule glycolysis and G-3-P synthesis, and bone FGF23 production with subsequent downregulation of Npt2a. Values are mean ± SEM. *P < 0.05, ***P < 0.0001. ANOVA with Tukey’s multiple comparisons test (**B**–**E**) or unpaired student’s *t* test (**F**).

Finally, we examined *Npt2a^–/–^* mice to reinforce the causal relationships among kidney-specific Pi uptake, kidney-derived G-3-P, and FGF23. In addition to wasting urinary Pi with resultant hypophosphatemia, these animals are known to have reduced FGF23 levels (33). Consistent with this, we found that basal levels of G-3-P were lower in *Npt2a^–/–^* mice relative to control mice. Further, we found that the increase in kidney cortex ^18^F-FDG signal observed in C57BL/6J mice following i.v. Pi was markedly attenuated in *Npt2a^–/–^* mice (Figure 7A and Supplemental Figure 7, A and B), and that neither i.v. Pi administration (Figure 7B) nor dietary Pi loading increased blood G-3-P levels if Npt2a was lacking (Figure 7, C and D). In turn, dietary Pi loading did not increase FGF23 levels in *Npt2a^–/–^* mice (Figure 7E). As with C57BL/6J and *Gpd1^–/–^* mice, however, exogenous G-3-P administration caused a significant increase in FGF23 levels in *Npt2a^–/–^* mice (Figure 7F), confirming that G-3-P was the key circulating factor — downstream of Pi-stimulated Gpd1 activity in the kidney proximal tubule — that stimulated FGF23 synthesis in bone.

## Discussion

Because FGF23 is produced by osteoblasts and osteocytes, the search for the Pi sensor has focused on bone. However, sensing within the kidney — and the proximal tubule specifically — is compelling for several reasons. First, the kidney is already the site of other sensors, including oxygen sensing upstream of erythropoietin production, as well as extensive expression of the CaSR, which plays a complementary role with calcium sensing in the parathyroid gland (36). Second, responsible for the reabsorption of Pi from the more than 140 liters of filtrate per day, the proximal tubule is uniquely poised to detect systemic Pi load. Third, the proximal tubule relies on oxidative lipid metabolism rather than glycolysis to meet its bioenergetic needs (37), in theory, making the glycolytic machinery available for other purposes. Fourth, the separation in time and space between Pi-stimulated G-3-P production in the kidney and downstream actions of G-3-P in bone shed light on the delay in the FGF23 response observed in experimental studies of Pi loading (9). Finally, because FGF23 reduces Npt2a expression, Pi sensing in the proximal tubule constitutes an elegant feedback loop whereby FGF23 action brakes its own synthesis by reducing Pi-stimulated G-3-P production (Figure 7G).

Phosphate is often considered in distinct domains, extracellularly in complex with calcium in bone, or inside cells as a mediator of signaling and bioenergetics. The direct entry of Pi into glycolysis — which contrasts with the vast majority of biochemical reactions that donate or receive phosphate as a high energy ester (ATP or GTP) — represents a potential point of convergence. Traditionally, the reaction catalyzed by Gapdh has been thought to lack significant regulation, largely based on low free–energy estimates for this reaction in erythrocytes. However, more recent studies have shown that variations in the concentrations of the reaction’s substrates and cofactors can alter the kinetics and shift the free energy of this reaction far from equilibrium (11, 12, 38). For example, intracellular Pi availability is believed to vary over several orders of magnitude and regulate Gapdh activity and overall glycolytic flux in yeast (13), but a specific role for Pi in regulating glycolysis in higher organisms has not been assessed.

Across primary human proximal tubule cells, OK cells, and primary mouse proximal tubule cells, we found that Pi was substrate limiting for glucose consumption and G-3-P production. Further, using ^18^F-FDG PET scanning, we demonstrated a striking increase in kidney glycolysis, with a concomitant decrease in urinary ^18^F-FDG excretion in response to exogenous Pi administration. The shapes of the PET scan curves are important and informative. In the bladder, the ^18^F-FDG signals increased steadily and then plateaued; that is, ^18^F-FDG accumulated in urine over time because it had nowhere else to go. By contrast, in the renal cortex, there was a discrete increase in ^18^F-FDG signal in Pi-treated animals — a change in the shape of the curves not observed in any other tissue — that lasted for approximately 20 minutes. Because the radioactive half-life of ^18^F-FDG is roughly 110 minutes, the increase and decrease in kidney cortex ^18^F-FDG signal over 20 minutes clearly indicates that the isotope and its phosphorylated derivative did not remain inside the kidney cells, but rather were returned to circulation. Thus, a difference of approximately 5% in ^18^F-FDG signal at any given time in the kidneys in response to Pi versus saline multiplies over time to a substantially greater difference in total accumulated ^18^F-FDG in the bladder. Consistent with our ^18^F-FDG PET data on Pi-stimulated glycolysis, i.v. Pi had no impact on circulating G-3-P levels in mice that were pretreated with empagliflozin or mice that had undergone bilateral clamping of the renal arteries and veins. The absence of Npt2a, which is only expressed in the kidney, also abrogated Pi-stimulated G-3-P production. Taken together, these findings support the conclusion that Pi-stimulated glycolysis is kidney-specific.

Notably, glycolysis is already utilized in the pancreatic beta cell for glucose sensing upstream of insulin secretion. Key features of this mechanism include a high capacity glucose transporter that permits rapid uptake regardless of extracellular concentration, along with low level expression of a hexokinase isoform whose activity is proportional to glucose availability; downstream metabolites reflective of glycolytic flux then control the rate of insulin synthesis and secretion (39). Whereas glycolytic flux and its stimulatory effect on insulin is circumscribed within the pancreas, numerous metabolites traditionally thought to function exclusively within the cell are now recognized to have extracellular signaling effects as well (40). Examples include succinate, short-chain fatty acids, aromatic amino acids, and even lactate; like G-3-P, lactate is a byproduct of glycolysis, which has been shown to bind a G protein coupled receptor on adipocytes to inhibit lipolysis (41). Thus, existing paradigms strongly support roles for glycolysis and its metabolites that extend well beyond ATP production alone.

Recently, G-3-P biosynthesis has been highlighted as a key pathway for cytosolic NAD+ regeneration conserved across yeast, worms, and mice (30). Here, we extend this paradigm to Pi-stimulated glycolysis, whereby Gapdh and Gpd1 activity are biochemically coupled through cytosolic NAD+, and the absence of Gpd1 abrogates Pi-stimulated G-3-P and FGF23 production. However, we note that Gpd1 does not provide the only route to G-3-P biosynthesis, and *Gpd1^–/–^* mice still have basal blood G-3-P levels that are approximately 70% the level of their *Gpd1^+/+^* littermates. Pi loading does not appear to alter expression of *Gpd1*, *Gpd2*, or *gk*, but more detailed studies are required to understand how these different enzymes contribute to systemic G-3-P metabolism, including fluctuations with fasting and feeding. Prior work on *Gpd2^–/–^* mice, which have reduced fertility and viability, is limited, and mice deficient in both Gpd1 and Gpd2 are extremely sick and die within a week of birth (42).

Npt2a and Npt2c are both primarily expressed in the kidney proximal tubule and both are downregulated by FGF23. Our intention is not to claim that only the former participates in kidney glycolytic Pi sensing. Rather, our experimental focus is on Npt2a because it plays a quantitatively greater role than Npt2c in kidney Pi reabsorption, enhancing our ability to elicit clear differences in Pi-stimulated glycolysis and G-3-P production with pharmacologic and genetic approaches. Because the proximal tubule is the predominant site of Npt2a expression, we believe it is quantitatively the major site of Pi-stimulated glycolysis and G-3-P production; however, we acknowledge that there is some low-level expression of Npt2a in other parts of the nephron. Finally, we note that other transporters mediate Pi uptake in other cell types throughout the body. Pit-1 is a widely expressed sodium-Pi cotransporter, and Pi deprivation upregulates its expression (43). This regulatory mechanism is also a version of Pi sensing, albeit at the individual cell level. Our findings on systemic Pi sensing upstream of G-3-P and FGF23 do not preclude other local mechanisms whereby cells may regulate their own Pi uptake according to cell-specific metabolic needs.

We note that Npt2a and Npt2c expression may not be the only features that facilitate kidney-specific Pi sensing. Sglt2 expression is restricted to the kidney proximal tubule, and we find that acute pharmacologic inhibition of this sodium-glucose cotransporter blocks Pi-stimulated G-3-P production in vitro and in vivo. With each successive positive clinical trial, SGLT2 inhibitors are gaining broader indications in diabetes, chronic kidney disease, and cardiovascular disease (44–46). Intriguingly, 2 studies have shown that SGLT2 inhibition increases both blood Pi and FGF23 levels (47, 48). This effect is likely multifactorial; in addition to blocking glucose reabsorption, SGLT2 inhibitors induce a natriuresis, increasing the electrochemical drive to reabsorb phosphate and causing a reduction in glomerular filtration rate. To what extent altered tubular cell G-3-P production might interact with these other factors warrants further study. More work is also required to understand the route by which G-3-P is released from proximal tubule cells. Organic anion transporters (OATs), which are highly expressed on the basolateral surface of the proximal tubule and share topology with an established bacterial G-3-P transporter, are attractive candidates for further investigation (49).

A substantial body of evidence has shown that the kidney proximal tubule has high gluconeogenic capacity, including classic microdissection studies demonstrating more gluconeogenic than glycolytic enzyme activity in the proximal tubule (50) as well as more recent work demonstrating the kidneys’ ability to maintain fasting euglycemia in mice lacking hepatic gluconeogenesis (51, 52). Our study shows that Pi stimulates glycolysis and G-3-P production, but primarily in the physiologically relevant context of feeding, when both glucose and Pi are ingested. This is entirely consistent with the proximal tubule’s contribution to gluconeogenesis during fasting, as well as the minor role that glycolysis plays in ATP production in these cells. Indeed, as already noted, it is precisely this minor bioenergetic role that makes proximal tubule glycolysis amenable to cooptation for another purpose like Pi sensing. That Pi-stimulated G-3-P and FGF23 production is biochemically coupled to glycolysis, in the fed state, suggests that this system developed to control Pi excretion over the course of hours following dietary ingestion. PTH acts much more rapidly, but, as noted, with the primary purpose of increasing and/or maintaining ionized calcium levels. In theory, this could be important immediately following a meal as the acute rise in ingested Pi could otherwise result in dangerous (arrhythmogenic) reductions in ionized calcium through calcium-Pi binding. Thus, our findings may shed light on the inherent logic of PTH and FGF23 physiology; more work is required, including more extensive human studies, to develop a comprehensive understanding of how these factors interact in different contexts.

In sum, our study identifies kidney proximal tubular cell glycolysis as a mammalian phosphate sensor, upstream of G-3-P and FGF23. In addition to addressing a fundamental gap in knowledge, these findings expand our view of the kidney as an endocrine organ, demonstrate a new paradigm whereby Pi availability can determine glycolytic rate and cytosolic NAD+/NADH in mammalian cells, and highlight potential targets for treating disorders of phosphate homeostasis that affect kidney, bone, and cardiovascular health.

## Methods

### Mice.

*C57BL/6J* and *CD-1* mice were purchased from the Jackson Laboratory and Charles River Laboratories. *Gpd1*-KO mice were generated within the MGH Endocrine Unit by delivering CRISPR genome engineering reagents directly to E0.7 CD-1 embryos via in situ electroporation (53). Guides were designed using GenScript CRISPR sgRNA Design Tool (https://www.genscript.com/) to target exon 2 of *Gpd1* with the following sequences: GGAAAGUGGACUAGGGUGAC and AACACGCAAAAGUGACUUCG. EZ crRNA and tracrRNA kits were obtained from Synthego, and Cas9 protein was purchased from PNA Bio. We confirmed that exon 2 was lacking in the kidney, muscle, and liver by PCR (Supplemental Figure 5A) and genomic sequencing (not shown). The following primers were used for genotyping: CAGGAGGTAAAAAAAAATCCAACAA and TGCCTCCTTCCCAGAGGAGGGGAGG. As determined by immunoblot, deletion of Gpd1 in the kidney may be associated with an increase in Gpd2 expression, but no change was observed for Gapdh (Figure 3B). To generate a model of inducible, mild hypoparathyroidism *Gpd1^–/–^* mice and *Gpd1^+/+^* littermates were crossed with *PTHcre-iDTR* mice that selectively expressed the DT receptor in parathyroid cells. *PTHcre* and *iDTR* primers have been previously described (23). *Npt2a* knockout mice were obtained from the Jackson Laboratory. Animals had been backcrossed onto the *C57BL/6J* background for more than 10 generations; genotyping by PCR amplification of genomic DNA was performed as previously described (54). Experiments used age-matched male mice at 8–16 weeks of age that were maintained in specific pathogen–free environments at 25°C and a 12-hour light/12-hour dark cycle.

### Cell lines and experiments.

Human Primary Renal Proximal Tubule Epithelial Cells (RPTEC) were purchased from ATCC. RPTEC were maintained in Renal Epithelial Cell Basal Medium (ATCC) supplemented with Renal Epithelial Cell Growth Kit (ATCC) and were incubated in 5% CO_2_/95% air at 37°C. OK cells were originally obtained from ATCC. Cells were maintained in DMEM: Nutrient Mixture F-12 (DMEM/F12, Thermo Fisher Scientific) supplemented with 10% FBS and 1% antibiotics (penicillin and streptomycin) and were incubated in 5% CO_2_/95% air at 37°C. Primary mouse proximal tubule cells were isolated as previously published (55). In brief, renal cortices were dissected and sliced into pieces. The fragments were transferred to collagenase and digested for 30 minutes. After digestion, the supernatant was sieved through 2 nylon sieves (pore sizes 250 m and 80 m). The proximal tubules were centrifuged, washed, and then resuspended into the appropriate amount of culture medium and plated. Cells were maintained in Renal Epithelial Cell Basal Medium supplemented with Renal Epithelial Cell Growth Kit and were incubated in 5% CO_2_/95% air at 37°C. Ocy454 cells were grown from a single subclone (56). Cells were passaged in α-MEM supplemented with 10% heat-inactivated FBS and 1% antibiotics (penicillin/streptomycin, Fungizone) at 33°C with 5% CO_2_. Cells were plated at 50,000 cells/mL and allowed to reach confluency at 33°C (typically in 2–3 days) prior to transfer to 37 °C. Experiments were performed on cells cultured at 37°C for 7 days.

### Animal experiments.

An equal mixture of monobasic and dibasic sodium phosphate (sodium Pi) 6 mg (Sigma-Aldrich) or sodium chloride 2.7 mg (Sigma-Aldrich) was dissolved in 100 μL of saline prior to i.v. injections. G-3-P 300mg/kg (Sigma-Aldrich) was dissolved in 500 μL of saline prior to i.p. injections. Empagliflozin 10 mg/kg (Selleck Chemical) and the Npt2a inhibitor PF-06869206 300 mg/kg (MedChemExpress) were administered by oral gavage. Recombinant human FGF23 (MedChemExpress) was dissolved in 100 μL of saline prior to i.p. injections. Serum was collected by retroorbital bleeding. For tissue collection following i.v. injections, mice were euthanized by cervical dislocation and tissues were collected, homogenized in water (1 mg:4 μL ratio), and then snap frozen in liquid nitrogen.

For mice that underwent clamping of renal arteries and veins, animals were placed on inhaled anesthesia on a temperature-controlled heating blanket and both renal pedicles were clamped with vascular clips (Roboz) (57). Sodium Pi or sodium chloride was then injected i.v. and serum was collected 10 minutes afterward.

To assess the acid-base impact of dietary Pi, C57BL/6J mice were acclimated in metabolic cages for 3 days and then transitioned to experimental diets (Envigo) containing normal Pi (0.6%), high Pi (1.2%), or normal Pi plus drinking water supplemented with 0.28 M NH_4_Cl for an additional 3 days. Urine samples were collected on days 1 and 3, blood was collected on day 3, and kidney tissue was collected after euthanasia on day 3. For assessment of mineral metabolism, *Gpd1^–/–^* and *Npt2a^–/–^* mice or their control littermates were fed a normal Pi and high Pi diet for 1 week and blood samples were collected on days 3 and 7. In a follow-up experiment in *Gpd1^–/–^* mice and *Gpd1^+/+^* littermates fed a high Pi diet, cinacalcet (Santa Cruz Biotechnology) 30 mg/kg per day in 0.5% methylcellulose (Abcam) was delivered via oral gavage daily for 7 days with blood samples collected on days 3 and 7. For induction of mild hypoparathyroidism with parathyroid specific expression of the DT receptor, 12 week old mice were injected with 5 μg/kg DT (Sigma Aldrich) i.p. on days 1 and 4, with baseline blood samples drawn on day 6. The mice were then transitioned from a normal Pi to high Pi diet for 5 days, followed by repeat phlebotomy on day 11. On the following day, mice were continued on a high Pi diet but also administered with i.p. G-3-P for 3 more days prior to phlebotomy and euthanasia on day 14.

### PET/MRI acquisition and analysis.

Animals were imaged in a 4.7 Tesla MRI scanner equipped with a PET insert (Bruker). Mice were anesthetized with isoflurane (4% for induction, 1%–1.5% for maintenance in medical air). After placement of a tail vein catheter for probe administration, mice were positioned in a prone position on a custom-built multi-animal cradle, which allows imaging up to 4 mice at a time. Animals were kept warm with an air heater system with the temperature and respiration rate monitored by a physiological monitoring system (SA Instruments Inc.) throughout the imaging session. The ^18^F-FDG PET probe (100–200 μCi) was mixed with sodium phosphate (6 mg) or sodium chloride (2.7 mg) and given i.v. as a bolus and followed by a 50 L saline flush. MRI and PET acquisition were performed simultaneously. Anatomic MR images were acquired with a 3D Fast Low Angle Shot (FLASH) sequence (repetition time (TR)/echo time (TE)/flip angle (FA) = 20 ms/3 ms/12°, field of view (FOV) = 86 × 65 × 50 mm, 0.25 mm isotropic resolution). Dynamic PET data were acquired in list-mode for up to 60 minutes after probe injection and were reconstructed using maximum likelihood expectation maximization (MLEM) algorithm with 12 iterations, 0.75 mm isotropic voxels, and binned into sequential time frames with durations of 9 × 20 seconds, 7 × 60 seconds, 6 × 300 seconds, 2 × 600 seconds. Reconstructed PET data were analyzed using AMIDE software package (58). Volumes of interest (VOIs) over renal cortex, bladder, liver, heart, and skeletal muscle were defined on MR images and used for quantifying radioactivity of each organ/tissue. Results were expressed as percentage of injected dose per cubic centimeter of tissue (%ID/cc).

### Human phosphate loading.

Three healthy people (2 men and 1 woman, aged 30–45 years) were recruited for this study, which consisted of 2 study visits separated by at least 2 weeks. All individuals provided written informed consent. During the first study visit (phosphate load), participants were asked to have breakfast 2 hours before the study visit, avoiding high-phosphate-containing foods; each person received 1.5 g of oral phosphate (K-Phos Neutral; Beach Pharmaceuticals) and underwent 6 blood draws, 1 before the oral loading and the remaining 5 done at 1-hour intervals after the oral loading. During the second visit (the control visit), the same people were asked to have breakfast 2 hours before the first collection, avoiding high-phosphate-containing foods, and subsequently underwent 6 blood draws at 1-hour intervals. Venous blood samples were collected in Lithium heparin-coated tubes and processed within 10 minutes of collection. Plasma was separated by centrifugation at 4°C and stored at –80°C until analysis.

### Blood mineral metabolism measurements.

Pi was measured using a colorimetric kit (Abcam), and iFGF23 and PTH were measured by the respective Immutopics ELISAs (catalog nos. 60-6800 and 60-2305) according to the manufacturer’s instructions. Colorimetric kits for calcium (Stanbio) and blood urea nitrogen (Invitrogen) were used, and 1,25(OH)_2_D was measured by enzymatic immunoassay (IDS), while creatinine was measured by liquid chromatography–mass spectrometry (LC-MS) as previously described (10).

### LC-MS measurements.

G-3-P was measured in 10 μL of plasma or tissue homogenate or 30 μL of cell culture media. In brief, samples were extracted with 90 μL (for plasma or tissue) or 70 μL (for culture media) acetonitrile/methanol (3:1 vv) containing 1 μg/mL G-3-P-^13^C_3_ (Cambridge Isotope Laboratories). The samples were centrifuged and 10 μL of supernatant was injected onto a 150 × 2.1 mm Atlantis HILIC Silica 3 μm Column (Waters). The column was eluted at a flow rate of 250 μL/min with 5% mobile phase A (10 mM ammonium formate in water) for 0.5 minutes, followed by a linear gradient to 40% mobile phase B (acetonitrile) over 8 minutes, then held at 40% mobile phase B for 5.5 minutes before returning to starting conditions. The peaks for G-3-P (transition 170.99/78.99) and G-3-P-^13^C_3_ (transition 173.99/78.99) were monitored in the negative-ion mode on a TSQ Quantiva Triple Quadrupole Mass Spectrometer (Thermo Fisher Scientific). Lactate and pyruvate were measured in 30 μL of tissue homogenate or cell culture media using hydrophilic interaction LC and multiple-reaction monitoring in the negative-ion mode on a 5500 QTRAP MS (SCIEX) as previously described (29).

### RNA-Seq.

mRNA obtained from the kidney cortex of C57BL/6J mice after 3 days on a high Pi (1.2%) or normal Pi (0.6%) diet were analyzed by RNA-Seq using the BGI DNBSEQ platform. A total of 17,457 genes were detected, and analysis of differentially expressed genes was conducted using DESeq2 (59), with a Q value (adjusted P value) threshold of less than 0.05. Complete data are deposited in GEO (accession GSE223176).

### Assessment of acid-base status.

Whole blood pH and PCO_2_ were assessed immediately after phlebotomy with an ABL800 FLEX analyzer (Radiometer) and [HCO_3_^–^] was calculated using the Henderson-Hasselbalch equation. Urine electrolytes were measured by IDEXX BioAnalytics. Quantitative PCR was performed on RNA extracted from kidney homogenates using a CFX Connect Real-Time PCR machine (Bio-Rad) and Cells Direct 1 Step qRT-PCR kit (Invitrogen) with the following primers: *Pck1*, forward GCATAACGGTCTGGACTTCT, reverse TGATGACTGTCTTGCTTTCG; and *Snat3*, forward CGAATCATGCCCACTGACAA, reverse AACCGCAGCGAAACAAAGG.

### Immunoblotting.

Tissue and cell lysates were prepared using RIPA buffer with protease inhibitors (Pierce, Thermo Fisher Scientific). Lysates (15–20 μg of protein) were separated by SDS-PAGE, and proteins were transferred onto nitrocellulose membranes. Membranes were blocked with 5% milk in TBS + 0.1% Tween20 and incubated with Gpd1 (sc-376219, Santa Cruz), Gpd2 (ab-188585, Abcam), Gapdh (ab-8245, Abcam), FLAG (F1804, Sigma Aldrich), or β-actin (8H10D10, Cell Signaling) antibodies overnight at 4°C. The next day, the membranes were washed and incubated with the appropriate HRP-coupled secondary antibodies (Invitrogen catalog numbers 31430 and 31460), and signals were detected with ECL (Pierce, Thermo Fisher Scientific). All immunoblots were repeated at least twice, with comparable results.

### Cell-based experiments.

To induce gluconeogenic conditions, OK cells were cultured in glucose and insulin free media (XF DMEM, Agilent) supplemented with lactate (10mM). A stable OK cell line expressing a TK-GAPDH was generated as follows. FLAG-TK-GAPDH plasmid was obtained from JM Orozco (18, 19). This plasmid was incorporated into lentiviral particles using psPAX2 (Addgene) and pMD2.G (Addgene) packing vectors in HEK293FT cells transfected with Lipofectamine 2000 (Thermo Fisher Scientific). The medium was changed the next day and collected 48 hours later. OK cells were exposed to lentiviral particles overnight in the presence of polybrene (2.5 μg/mL), and TK-GAPDH–expressing cells were selected in media containing puromycin (4 μg/mL).

Cells were treated with an equal mixture of monobasic and dibasic sodium Pi (Sigma-Aldrich) or sodium sulfate (VWR), with or without 1 μM empagliflozin (Selleck Chemical), 25 mM 2-DG (Sigma Aldrich), 5 μM CGP 3466 (ChemCruz), 10 mM 1-thioglycerol (TCI America), 33 μM koningic acid (Adipogen Life Sciences), 1 mM each dihydroxyacetone phosphate/glyceraldehyde-3-phosphate (Sigma Aldrich), or 1 mM AKB (Sigma Aldrich); 0.5 μM rotenone (Sigma Aldrich); 0.1 μM PTH (Sigma Aldrich); or 10 μM PF-06869206 (MedChemExpress). For experiments testing the effect of different media glucose concentrations, low-glucose DMEM (Thermo Fisher Scientific) containing 5.5 mM glucose was used, with additional glucose added to achieve final concentrations of 17.5 mM or 50mM as needed. For the experiment testing the effect of different media calcium concentrations, DMEM (normal calcium, 20 mg/dL) was compared to MEM Hank’s Balanced (low calcium, 14 mg/dL; Thermo Fisher Scientific). For all other experiments, basal media for each cell line was used. 1 × Pi and sulfate = 0.9 mM. Cells were preincubated with empagliflozin and 2-DG for 30 minutes, PTH for 4 hours, and with all other compounds for 10 minutes before the addition of sodium Pi or sodium sulfate.

### Glucose consumption.

Media glucose consumption was measured with a glucose colorimetric/fluorometric assay kit according to the manufacturer’s instructions (BioVision).

### Measurement of cytosolic NAD+/NADH ratio using SoNar.

OK cells were seeded at 600,000 cells in 10 cm culture dishes and incubated overnight. The following day, media was replaced with fresh antibiotic-free DMEM and cells were transfected with Fugene HD transfection reagent. More specifically, OK cells were incubated with 5 mg of pCDNA3.1-SoNar (provided by Y. Yang and J. Loscalzo) and 18 mL of Fugene HD transfection reagent for 24 hours, then transfected cells were resuspended with trypsin and replated at 20,000 cells per well on a 96-well clear bottom black microplate (Corning) in FluoroBrite DMEM (Life Technologies) containing 5% dialyzed FBS, 1 mM pyruvate and 2 mM L-glutamine. Twenty-four hours after transfected cells had been seeded on the 96-wells plate, cells were washed once with 100 mL of FluoroBrite DMEM and media was changed to 100 mL of FluoroBrite DMEM containing 2% dialyzed FBS and 2 mM L-glutamine. 1 hour later, the following compounds were added: DMSO (for a final concentration of 0.1%), rotenone (0.5 μM), Pi (1 ×, 3 ×, and 10 ×), AKB (1 mM), or Pi (1 ×, 3 ×, and 10 ×) + AKB (1 mM). 1 × Pi = 0.9 mM. Dual-excitation ratios of the SoNar sensor were measured immediately afterward and at the specified time points using an Envision multi-label reader (PerkinElmer) with 430 nm or 485 nm excitation and 535 nm emission for both excitation wavelengths.

### Seahorse.

Extracellular acidification rates (ECARs) and oxygen consumption rates (OCRs) of live OK cells treated with supplementary Pi ± empagliflozin was measured by a Seahorse XFe96 Analyzer (Agilent) according to the manufacturer’s instructions.

### Statistics.

2-tailed student’s *t* tests or 2-way ANOVAs were used to determine significance; details are provided in the figure legends. Statistical analyses were performed using Graph Pad Prism 6. *P* < 0.05 was considered significant, except for RNA-Seq data, where significance was set at Q value (*P*_adj_) < 0.05.

### Study approval.

All animal studies were approved by the Institutional Animal Care and Use Committee of the Massachusetts General Hospital and conducted under their guidelines. The human phosphate loading study was approved by the IRB at Mass General Brigham (Boston, Massachusetts, USA).

## Author contributions

WZ and PS carried out most of the experiments and analyzed the results. IYZ and PC performed PET/MR image acquisition and analysis. XVP conducted the human pilot study and, along with OLW, carried out several cell culture experiments. DW assisted with mouse surgery and tissue harvests. MS, MM, and TK created the Gpd1 knockout mouse. KAP and CBC performed LC-MS analyses and contributed LC-MS methodologic expertise. MM, MNW, and HJ contributed to project conception, contributed hypoparathyroid and Npt2a knockout mouse models, and analyzed results. EPR designed the study, analyzed results, and wrote the manuscript with input from all authors. Authorship order for the co–first authors was determined based on mutual agreement.

## Supplementary Material

Supplemental data

## Version 1 02/23/2023

In-Press Preview

## Version 2 04/17/2023

Electronic publication
