# Supplementary material for: Kidney glycolysis serves as a mammalian phosphate sensor that maintains phosphate homeostasis
Source: J Clin Invest. 2023 Apr 17;133(8):e164610. doi: 10.1172/JCI164610 (PMC10104895; doi:10.1172/JCI164610)
Supplement: Supplemental data [file jci-133-164610-s118.pdf]

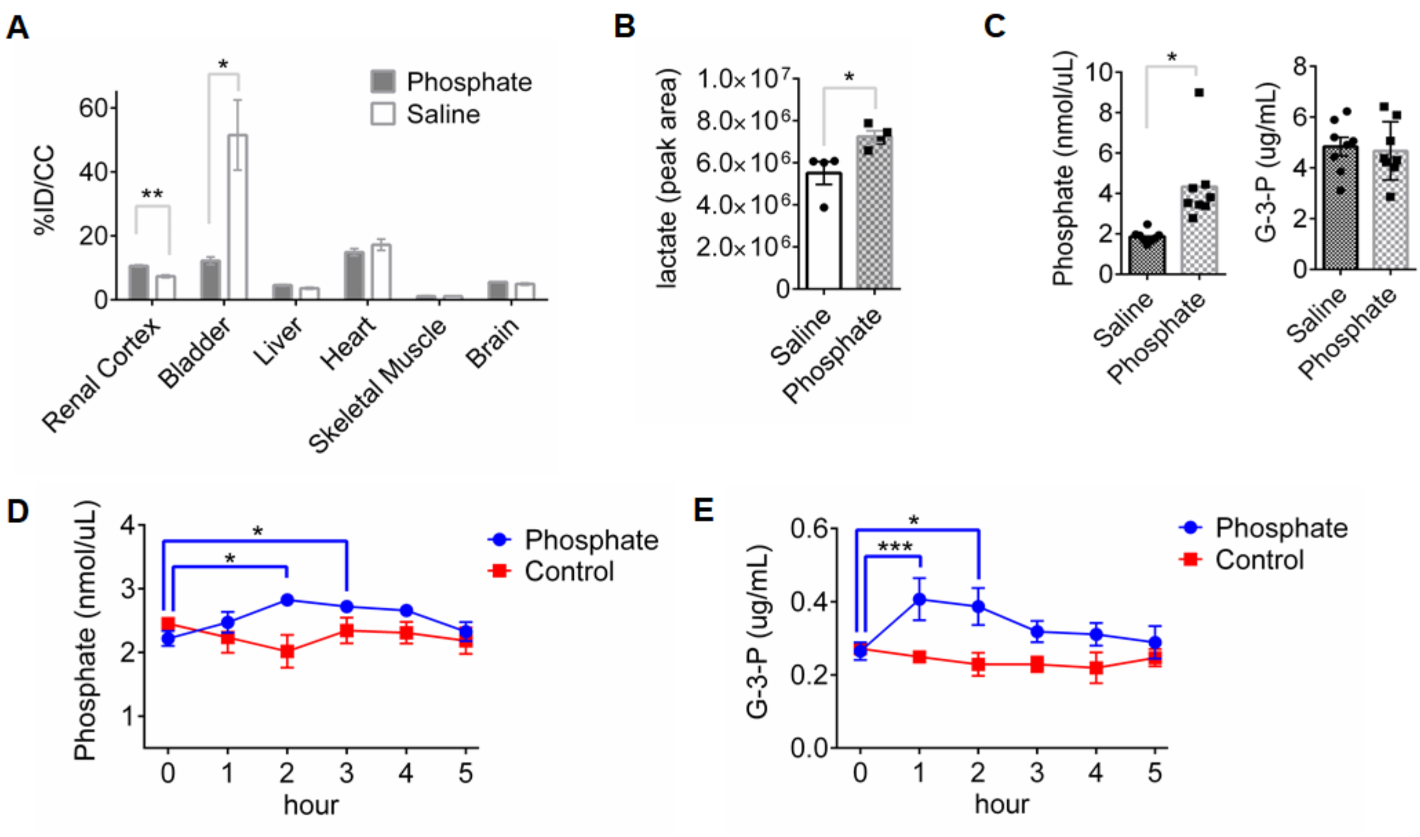

Supplemental Figure 1. Phosphate-stimulated G-3-P production is kidney-dependent and occurs in humans. (**A**) ^{18}F-FDG signal expressed as percent injected dose per cubic centimeter of tissue (%ID/CC) in renal cortex, bladder, liver, heart, skeletal muscle, and brain 10 min after i.v. ^{18}F-FDG and either sodium phosphate or sodium chloride in C57Bl/6J mice (n=4 per group). (**B**) Kidney lactate levels 10 min after i.v. sodium phosphate or sodium chloride (n=4 per group). (**C**) Blood phosphate and G-3-P concentrations 10 min after i.v. sodium phosphate or sodium chloride in C57Bl/6J that underwent bilateral renal artery and vein clamping (n=8 per group). (**D, E**) Blood phosphate (D) and G-3-P (E) concentrations in human subjects following phosphate (1.5g oral ingestion) or control (no ingestion) (n=3 per group). Values are means ± s.e.m. *P<0.05, **P<0.001, ***P<0.0001, unpaired t-test with Holm-Sidak method (A), unpaired student's t-test (B,C), or ANOVA with Dunnett's multiple comparisons test (D, E).

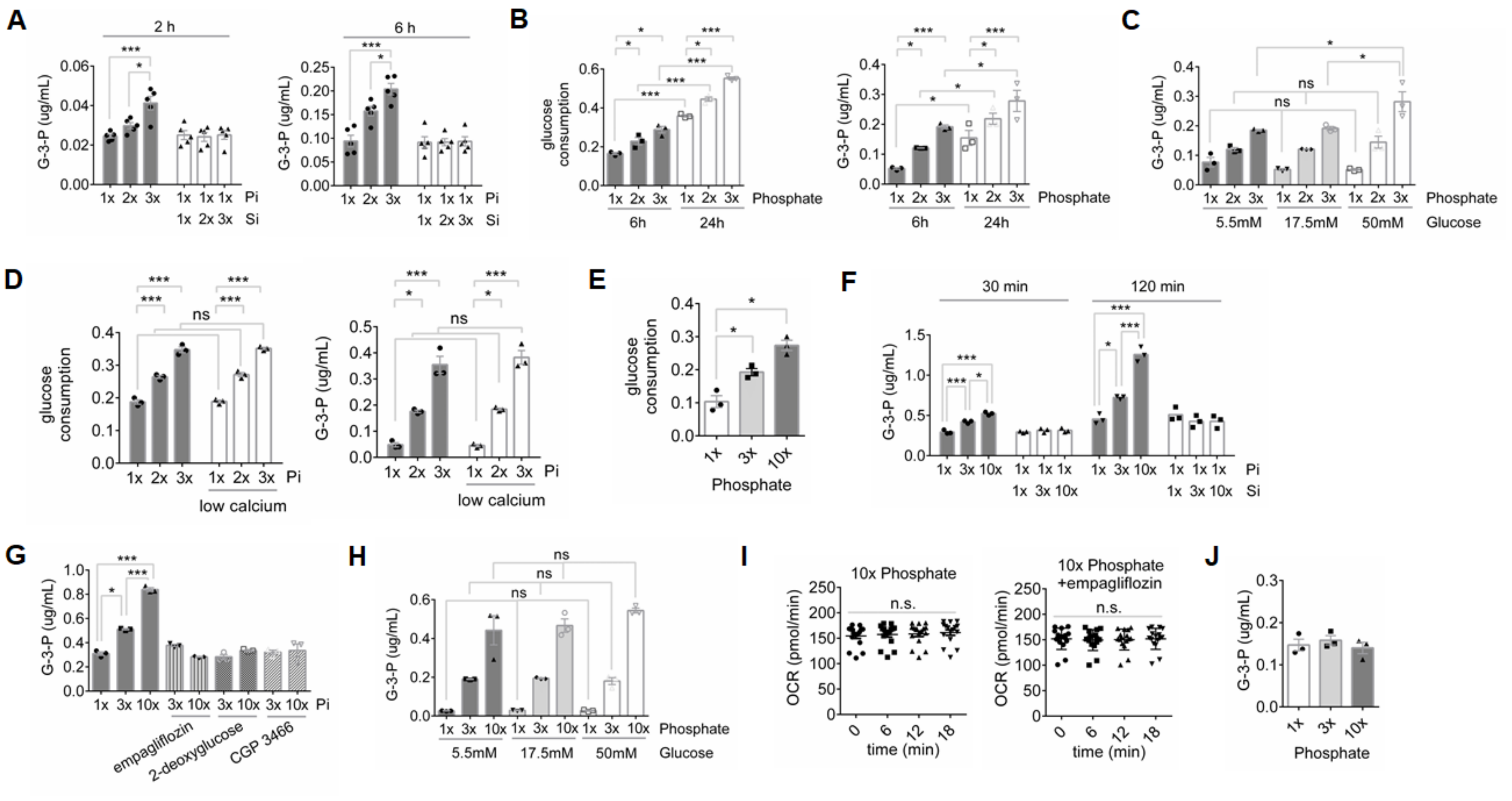

Supplemental Figure 2. Phosphate stimulates glycolysis and G-3-P production in human proximal tubule cells and OK cells. (**A**) Media G-3-P concentration at 2 hr and 6 hr from primary human proximal tubule cells incubated with increasing concentrations of sodium phosphate (Pi) or sodium sulfate (Si) (n=5 per group). (**B**) Media glucose consumption (left) and G-3-P concentrations (right) at 6hr and 24hr for primary human proximal tubule cells incubated with increasing concentrations of Pi (n=3 per group). (**C**) Media G-3-P concentrations at 6hr for primary human proximal tubule cells incubated with increasing concentrations of Pi in 5.5mM, 17.5mM, and 50mM glucose media (n=3 per group). (**D**) Media glucose consumption (left) and G-3-P concentrations (right) at 6hr for primary human proximal tubule cells incubated with increasing concentrations of Pi in normal calcium (20 mg/dL) or low calcium (14 mg/dL) media (n=3 per group). (**E**) Media glucose consumption at 120 min from OK cells incubated with increasing concentrations of Pi (n=3 per group). (**F**) Media G-3-P concentration at 30min and 120 min from OK cells incubated with increasing concentrations of Pi or Si (n=3 per group). (**G**) Media G-3-P concentration at 120 min from OK cells incubated with increasing concentrations of Pi ± empagliflozin (1µM), 2-deoxyglucose (25mM), or CGP 3466 (5µM) (n=3 per group). (**H**) Media G-3-P concentrations at 2hr for OK cells incubated with increasing concentrations of Pi in 5.5mM, 17.5mM, and 50mM glucose media (n=3 per group). (**I**) Oxygen consumption rate (OCR) measured by Seahorse in OK cells treated with 10x Pi ± empagliflozin (1µM) (n=16 per group). (**J**) Media G-3-P concentration at 2 hr from cultured osteocytes (Ocy454 cells) incubated with increasing concentrations of Pi (n=3 per group). 1x Pi and Si = 0.9mM. Values are means ± s.e.m. *P<0.05, ***P<0.0001. ANOVA with Tukey's multiple comparisons test.

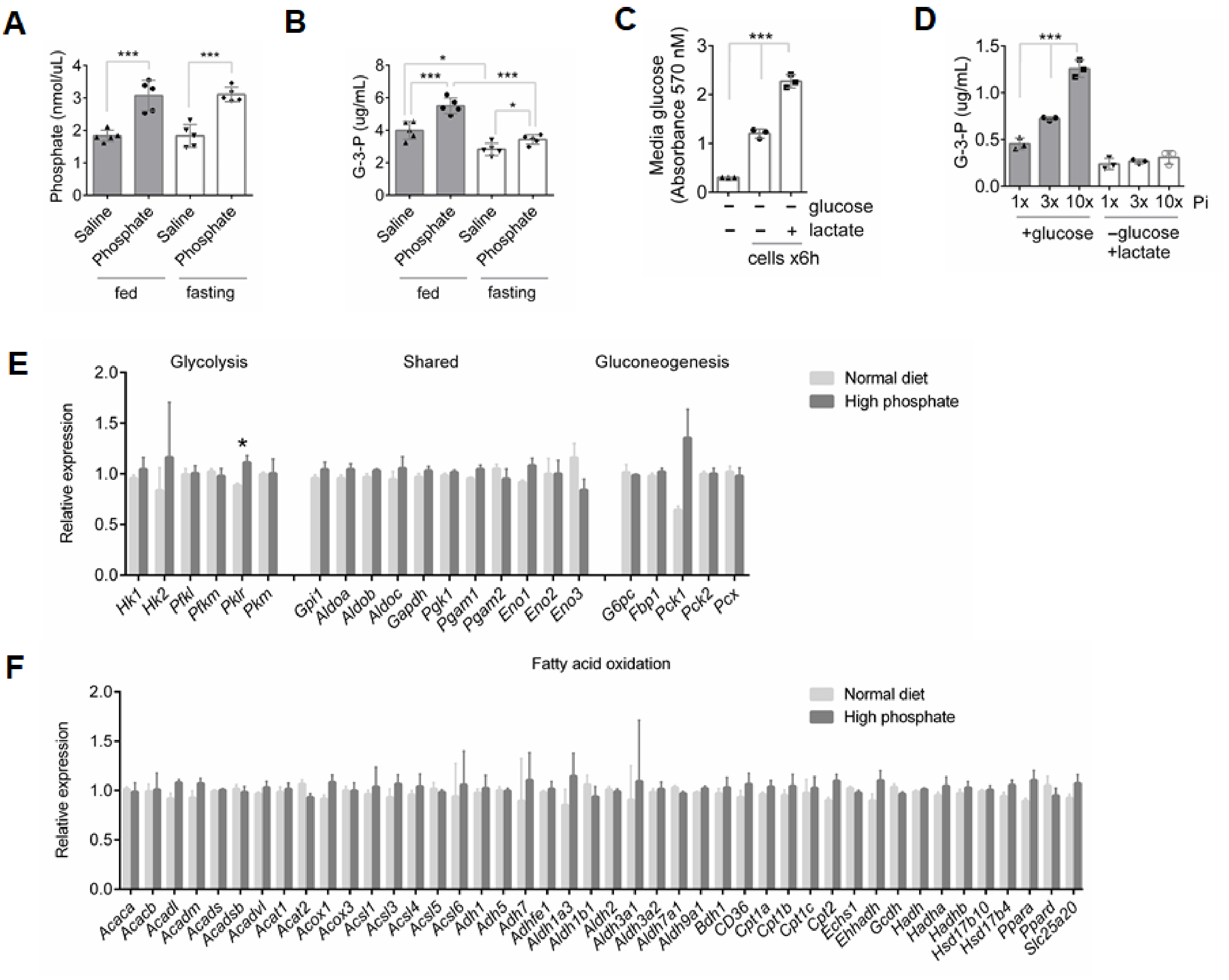

Supplemental Figure 3. Phosphate stimulates G-3-P production in the fed state, without systematic changes in metabolic gene expression. (**A,B**) Blood phosphate (A) and G-3-P (B) concentrations 10 min after i.v. sodium phosphate or sodium chloride in fed and fasted (12 hr) C57Bl/6J mice (n=5 per group). (**C**) Glucose in media alone and after 6 hr on OK cells incubated with glucose free media ± lactate 10mM. (**D**) Media G-3-P from OK cells treated with increasing concentrations of phosphate (Pi) under glycolytic or gluconeogenic conditions for 6 hr. 1x Pi = 0.9mM. Values are means ± s.e.m. *$P<0.05$, ***$P<0.0001$. Unpaired student's t-test (A,B) or ANOVA with Tukey's multiple comparisons test (C,D). (**E,F**) Relative expression of genes involved in glycolysis and gluconeogenesis (E) and fatty acid oxidation (F) in kidney cortex of C57Bl/6J mice after 3 days on a high Pi (1.2%) or normal Pi (0.6%) diet (n=4 per group). *Q-value ≤ 0.05.

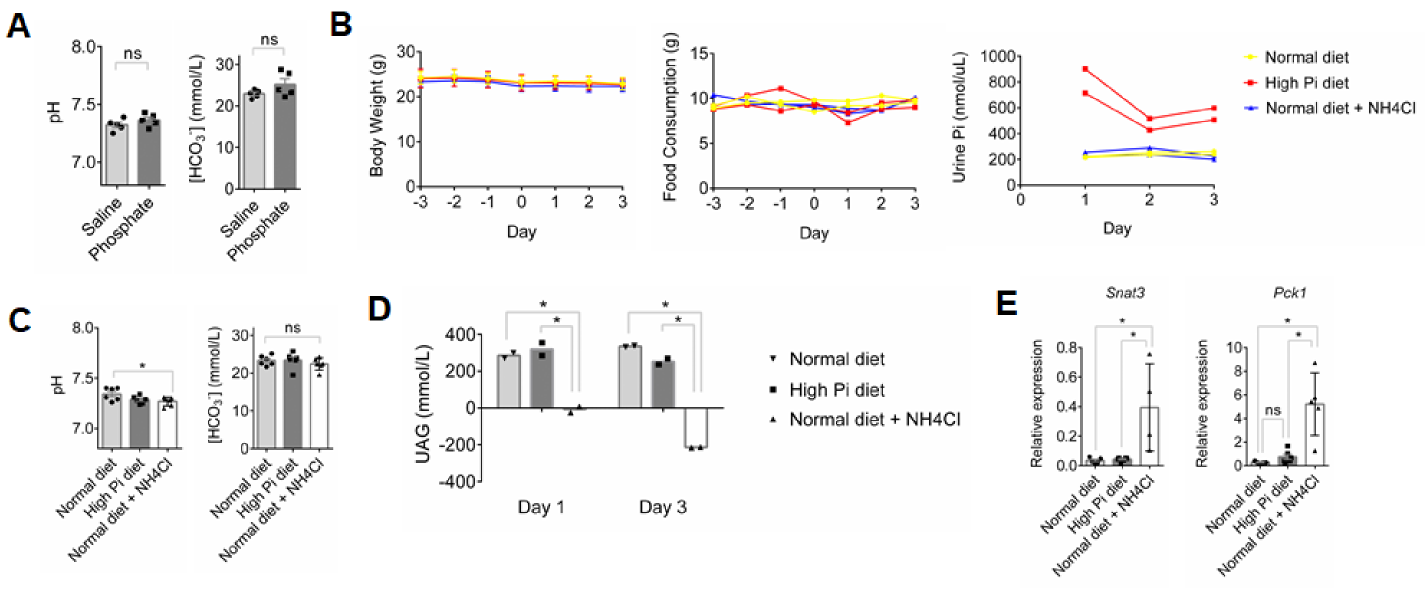

Supplemental Figure 4. Supplemental sodium phosphate does not induce metabolic acidosis. (**A**) Blood pH and bicarbonate concentrations in C57Bl/6J mice 10 min after i.v. sodium phosphate (6mg) or equimolar sodium chloride (2.7mg) (n=5 per group). (**B-E**) Daily body weight, food consumption, and urine phosphate (B), blood pH and bicarbonate concentrations (C), urine anion gap (UAG) (D), and kidney cortex *Snat3* and *Pck1* expression (E) in C57Bl/6J mice acclimated for 3 days (-3 to 0) and then maintained on normal diet (0.6% Pi) or transitioned to either high Pi (1.2%) diet or normal diet plus 0.28M NH_4Cl drinking water for 3 days (0 to 3) (n=6 per group; note food consumption, urine phosphate, and UAG were assessed per metabolic cage, n=2 cages of 3 mice per diet group). Values are means ± s.e.m, or ± s.d. for body weight (B). *P<0.05. Unpaired student's t-test (A) or ANOVA with Tukey's multiple comparisons test (C-E).

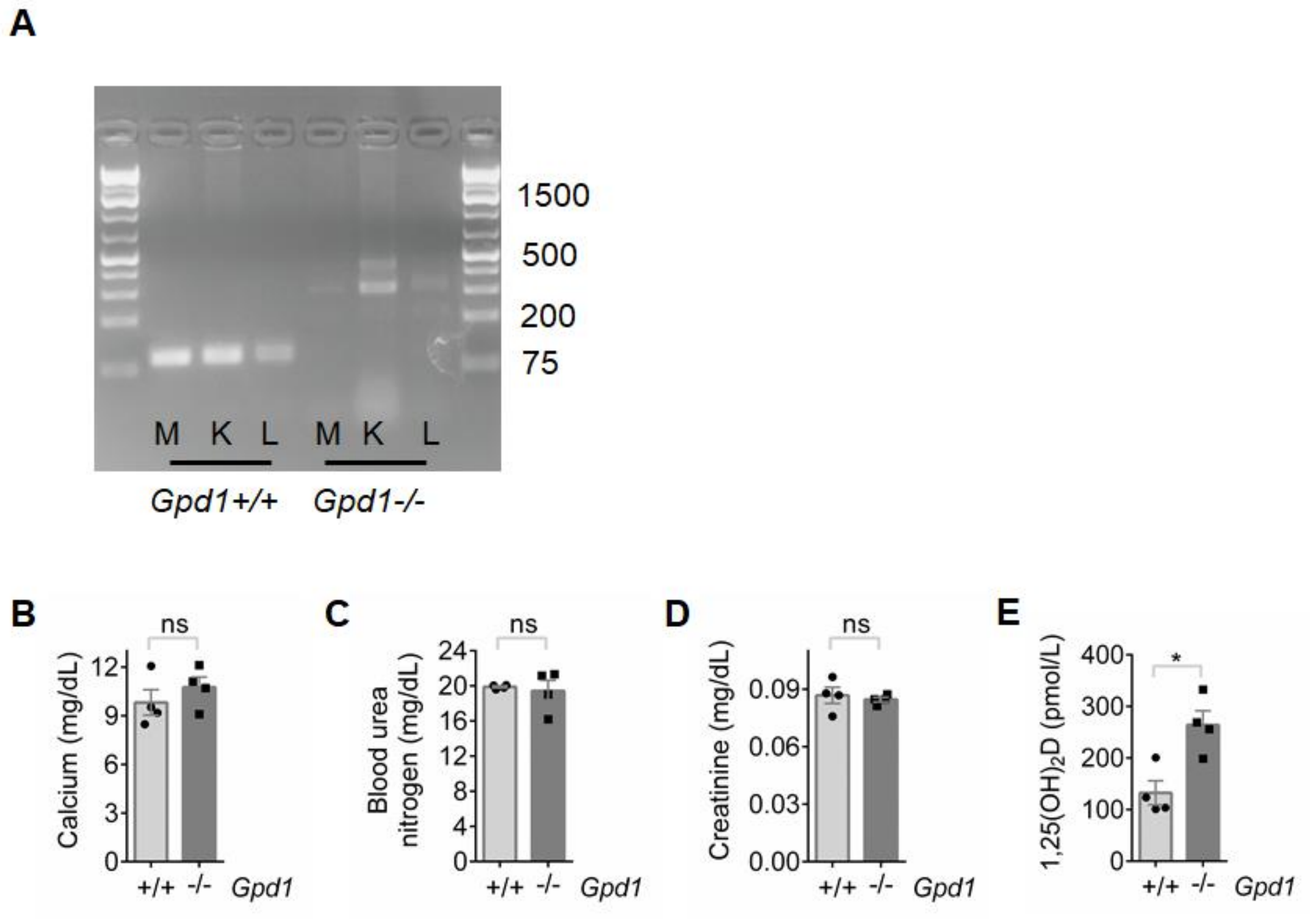

Supplemental Figure 5. *Gpd1* deletion. (**A**) PCR showing 134bp product of *Gpd1* exon 2 in muscle (M), kidney (K), and liver (L) tissue. (**B-E**) Blood calcium (B), urea nitrogen (C), creatinine (D), and $1,25(OH)_2D$ (E) levels in *Gpd1+/+* and *Gpd1-/-* mice on a normal diet (n=4 per group). Values are means ± s.e.m. **P*<0.05. Unpaired student's t-test (B-E).

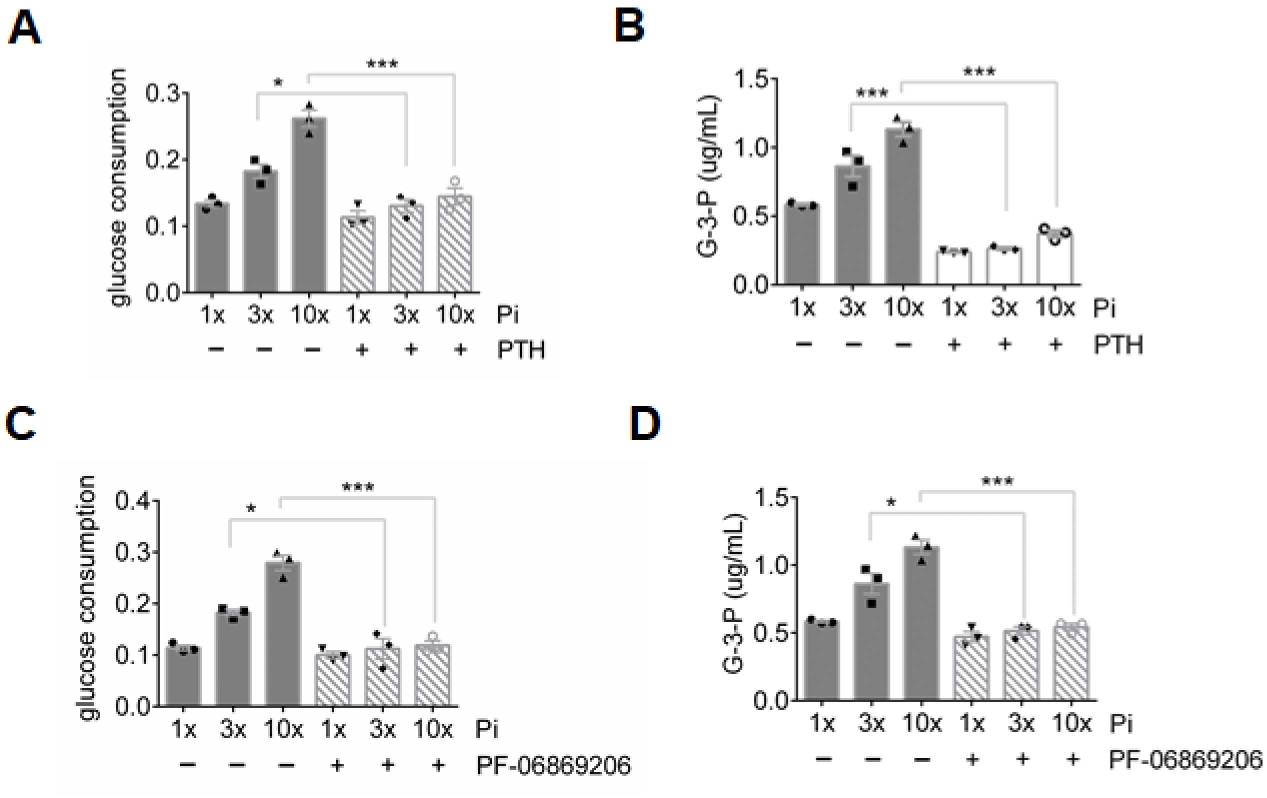

Supplemental Figure 6. Npt2a inhibition abrogates phosphate-stimulated glycolysis and G-3-P production in vitro. (**A,B**) Media glucose consumption (A) and G-3-P concentration (B) from OK cells treated with increasing concentrations of phosphate (Pi) ± PTH (0.1µM) for 2 hr (n=3 per group). (**C,D**) Media glucose consumption (C) and G-3-P concentration (D) from OK cells treated with increasing concentrations of phosphate ± PF-06869206 (10µM) for 2 hr (n=3 per group). 1x = 0.9mM. Values are means ± s.e.m. *P<0.05, ***P<0.0001. ANOVA with Tukey's multiple comparisons test.

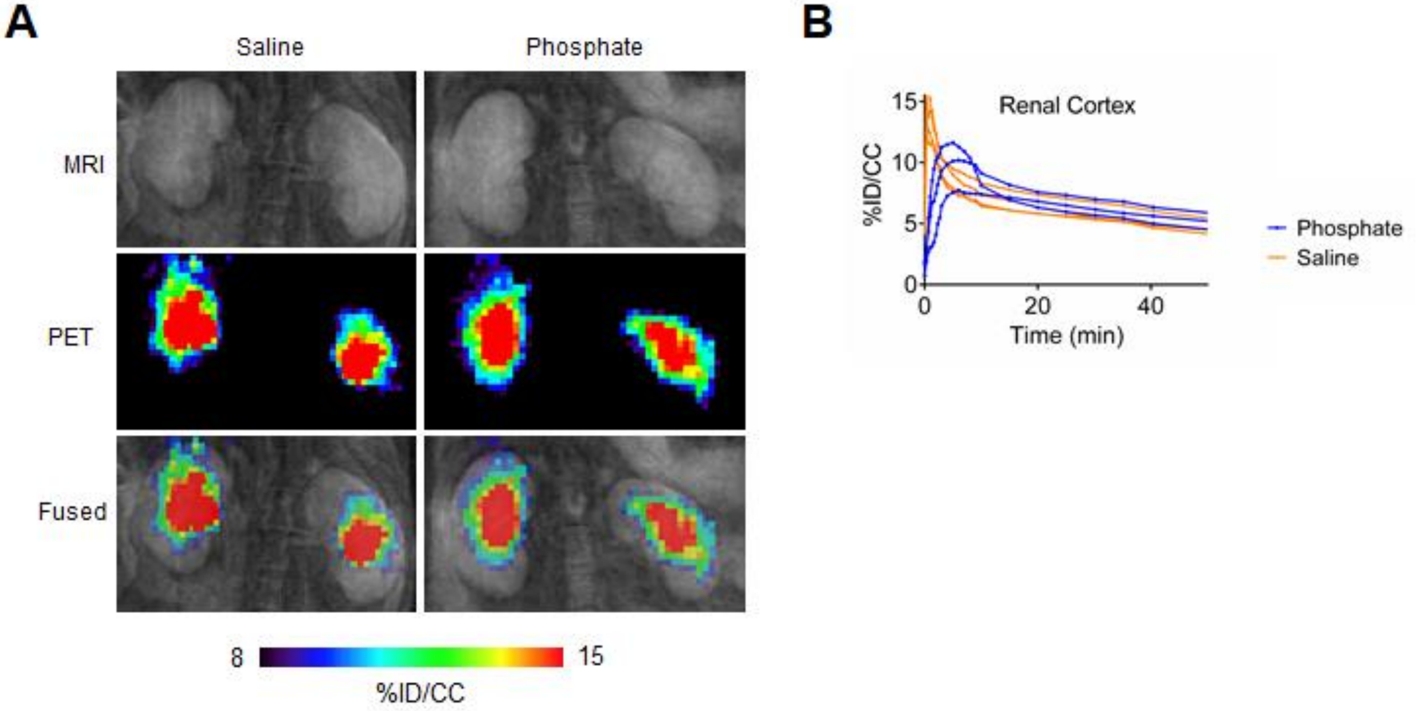

Supplemental Figure 7. Phosphate-stimulated glycolysis is attenuated in *Npt2a-/-* mice. (**A**) Representative kidney coronal MRI, PET, and fused images 10 min after i.v. ^{18}F-FDG and either sodium phosphate or sodium chloride in *Npt2a-/-* mice. (**B**) ^{18}F-FDG signal expressed as percent injected dose per cubic centimeter of tissue (%ID/CC) in renal cortex following i.v. ^{18}F-FDG and either sodium phosphate or sodium chloride in *Npt2a-/-* mice (n=3-4 per group).
